# Patella height ratios diagnose the same healthy knees differently

**DOI:** 10.1038/s41598-024-83663-2

**Published:** 2025-01-02

**Authors:** Martinique Vella-Baldacchino, Alessandra Cipolla, Zahid Asghar, Sally LiArno, Ahmad Faizan, Jean-Noel Argenson, Matthieu Ollivier

**Affiliations:** 1 Department of Surgery & Cancer, MSk Lab – Imperial College London, Sir Michael Uren Hub, 86 Wood Ln, London, W12 0BZ UK; 2 Department of Orthopedics and Traumatology, Institute of Movement and Locomotion, St. Marguerite Hospital, 270, boulevard Sainte- Marguerite, BP 29, Marseille, 13274 France; 3 University of Turin, CTO Hospital (C.T.O. Centro Traumatologico Ortopedico), Via Gianfranco Zuretti, 29, Torino, 10126 TO Italy; 4 University of Lincoln, Lincoln Medical School, LinCTU, Lincolnshire, LN6 7TS UK; 5 Stryker Orthopaedics, Mahwah, NJ USA

**Keywords:** Knee, Patella, Patellofemoral, Patella height ratio, Insall-salvati, Caton-deschamps, Epidemiology, Bone

## Abstract

Our study aims to investigate if the ratios proposed by Insall-Salvati and Caton-Deschamps follow the theory of normal distribution levels in a healthy population. 434 skeletal mature, healthy **knees** were obtained from a CT-scan-based modelling system (SOMA). Patellae height ratios were measured using the Insall-Salvati ratio and the Caton-Deschamps index. The patella height ratios of the sample population were plotted on a quantile plot and diagnosed as patella alta, baja or normal using the original ratio definitions. The study population patella height values at the 95th, 98th and 5th centiles were identified and compared to those described as alta or baja by Insall-Salvati and Caton-Deschamps. This meant that **if the patient had patella alta**,** this would be defined as a ratio of ≥ 1.2 and hypothesised that this would align at the 98th centile of the study population**,** whilst if diagnosed as patella baja (≤ 0.74)**,** this should align at the 5th centile of the population.** The inter-rater reliability of both ratios was calculated using kappa statistics. **Two authors made all calculations and compared them for consistency using the intraclass correlation coefficient.** For the Insall-Salvati ratio, the study population’s value at the 98th centile was equivalent to what Insall-Salvati describes as alta, a ratio of ≥ 1.2. In the study population, patients are overdiagnosed if using the Insall-Salvati ratio as patella baja ≤ 0.74, as the value at the 5th centile in the study population was 0.59. Using the Caton-Deschamps index, the 95th centile was 1.3, higher than the patella alta ratio determined by Caton as a ratio ≥ 1.2. Using Caton-Deschamps, patients are being underdiagnosed if utilising a patella baja ratio of ≤ 0.6. In our population, the 5th centile was 0.75. The two ratios had a kappa coefficient of 0.01, which indicates poor inter-rater reliability. Depending on the ratio used, caton-Deschamps and Insall-Salvati ratios diagnose patients as alta or baja differently. The current patella height ratios have originated from very small, select population samples. The future of patella height discussion should begin with data representing large populations. Then, we can discuss the upper and lower limits of abnormality.

**Supplementary Information:**

The online version contains supplementary material available at 10.1038/s41598-024-83663-2.

## Introduction

The position of the patella in the sagittal plane is referred to as patella height. The patella may be classified as patella alta or baja. Various measures of patella height have been proposed, including the Caton-Deschamps ratio, the Blackburne-Peel index or the modified Insall-Salvati, and the patellotrochlear index (PTI)^1–4^. However, in the literature, there is still no generally accepted consensus. The Insall-Salvati index has been criticised because of the high evaluator variability and the difficulty of obtaining a true lateral knee profile on the radiographs however, it remains the most widely used. The 1971 original study was based on a normal distribution of 114 pathological knees^4^.

Patella alta is defined as a high-riding patella in relation to the trochlear groove of the femur^5^. This condition can cause an abnormal and insufficient patella engagement in the trochlea groove and increase the risk of subluxation or dislocation^6^. Patella baja is defined as a patella that is too distal to the femoral trochlea due to patella tendon shortening, which may happen after any surgery involving the upper tibia^7^. The condition causes the patella to always be in contact with the trochlea in extension, in contrast to the normal patella^8^. This causes a restricted range of movement, crepitation and retropatellar pain^9^. The definition of patella alta or baja strictly depends on the measurement method used.

Our study aims to investigate if the ratios proposed by Insall-Salvati and Caton-Deschamps follow the theory of normal distribution levels in a healthy population. This means if patella alta is defined as a ratio of ≥ 1.2, this should align with the 98th centile of the study population, whilst if diagnosed as patella baja (≤ 0.74), this should be at the 5th centile of the population^4^. With respect to Caton-Deschamps, the aim is to determine if a healthy population has patella alta at the 95th centile with a ratio of ≥ 1.2 or patella baja at ≤ 0.6^1,10^.

## Methods

All scans were obtained per the local legal and regulatory requirements, which included ethics board approval and informed patient consent from all subjects and/or their legal guardians. This study was conducted following approval of the research protocol by the local ethical committee (Aix-Marseille University) and performed following relevant guidelines and regulations. The research was carried out in compliance with the Helsinki Declaration. In this **retrospective observational population cohort study**, the Stryker Orthopaedics Modeling and Analytics (SOMA) system (Stryker, Mahwah, New Jersey, USA) was queried for all patients who had available measurements of tibial tuberosity torsion. The SOMA database was developed using a global sample of over 3,600 patients who received CT scans with their knee in a semi-extended position (**10º flexion**) and three-dimensional (3D) bone models for medical indications such as CT angiography (70%), polytrauma (20%) and other reasons (10%)^11^. **SOMA automatically transfers measurements defined on an averaged 3D-bone template based on the available 1070 datasets to each dataset**^12,13^. **This ensures highly accurate and reproducible measurements across a large population**^12,13^.

Patients without measurements of patellar height **using the semi-automated method described above were excluded from the analyses for reasons such as amputations because of polytrauma**. Demographic variables including age, sex, height, weight, body mass index (BMI), and ethnicity, were obtained from the database along with the radiographic measurements.

A total of 434 skeletal mature healthy **knees** were retrieved from a CT scan-based modelling and analysis system (SOMA, Stryker, Mahwah, New Jersey)^12^. Basic patient demographics were reported as seen in Table 1. Qualitative variables were reported as a number and quantitative variables as mean ± standard deviation.

**Table 1:** Study population cohort characteristics.

Parameter	Summary Statistic
Number of patients (knees)	434
Female (number of patients)	207
**Age**	
Male - Mean (SD)	59 (17)
Female - Mean (SD)	59 (16)
**Body Mass Index (kg/m2)**	
Male - Mean (SD)	25 (4)
Female - Mean (SD)	25 (5)
**Ethnic group**	
African	5
Asian	186
Caucasian	235
Middle Eastern	8

**Fig. 1:**
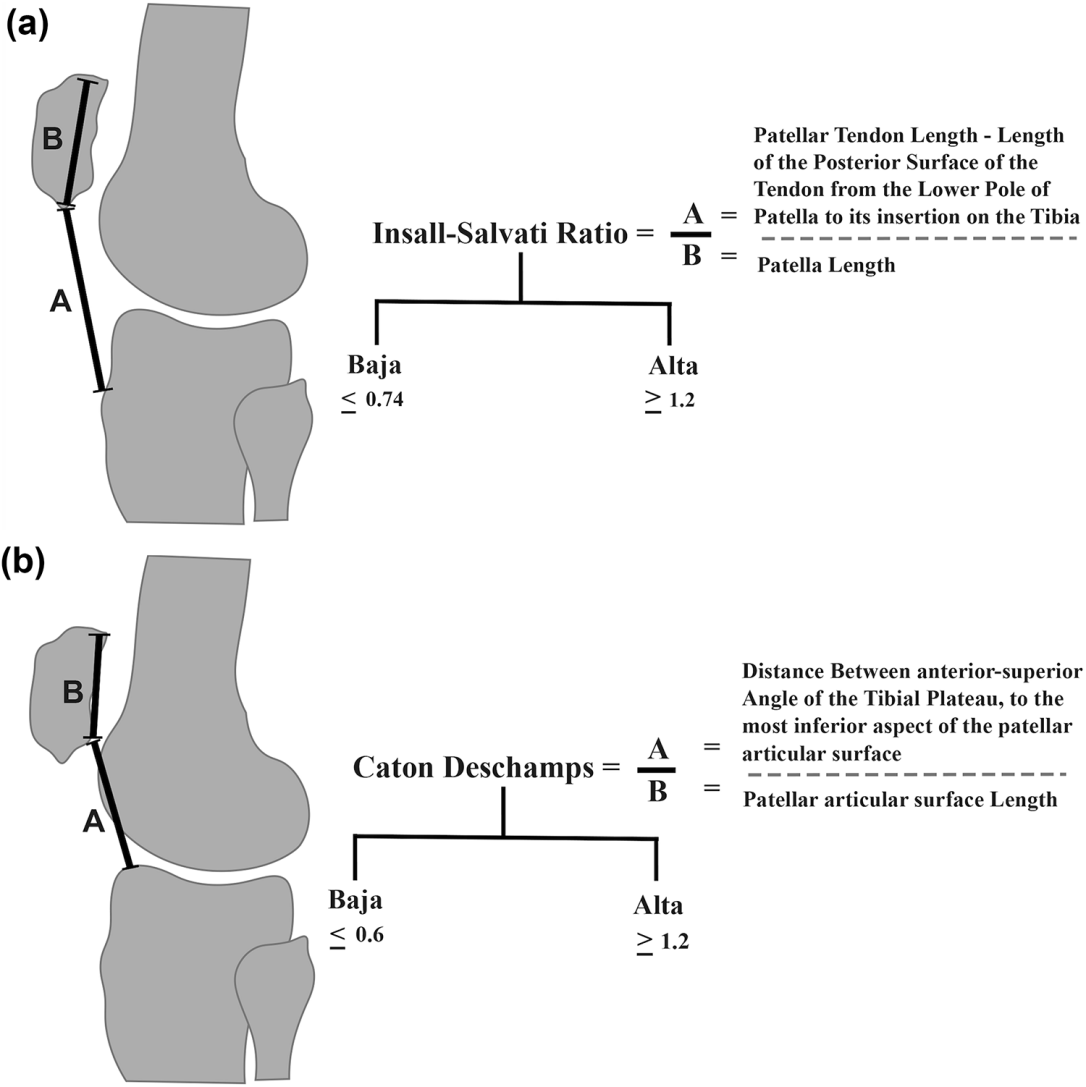
(**a**) Illustration depicting calculation of Insall-Salvati Ratio. (**b**) Illustration depicting calculation of Caton-Deschamps Ratio.

For each patient, the sagittal CT images were then analysed to measure the patella height using Insall – Salvati ratio and the Caton – Deschamps index. The Insall- Salvati index was calculated using the ratio of the patella tendon length to the length of the patella (Fig. 1a)^5^. Caton- Deschamps ratio was calculated using the ratio of the length of the patella articular surface to the distance from the anterior angle of the tibia plateau (Fig. 1b)^11^. **All calculations were made by two authors separately and compared for consistency using intraclass correlation coefficient.**

As seen in Table 2, using Insall – Salvati and Caton – Deschamps’ original papers, palta alta was defined if the ratios were ≥ 1.2^4,10^. Patella baja was defined ≤ 0.74 according to Insall-Salvati ratio and ≤ 0.6 with Caton – Deschamps ratio^4,10^.

**Table 2:** Definitions of patella alta and patella baja according to the original papers.

Caton- Deschamps index^5,6^		Patella Alta	Patella Baja
Definition	Distance between the anterior-superior angle of the tibial plateau, to the most inferior aspect of the patellar articular surface (A)	A/B ≥ 1.2	A/B ≤ 0.6
	Patella articular surface length (B)		
Number of patients	141 (80 male, 61 female)		128

Insall-Salvati Ratio^15^		Patella Alta	Patella Baja
Definition	A: patellar tendon length - length of the posterior surface of the tendon from the lower pole of the patella to its insertion on the tibia	A/B ≥ 1.2	A/B ≤ 0.74
	B: patella length	Length of the patellar tendon should not differ from that of the patella by more than 20%	
Number of patients	114	16	3

Each patient’s patella height was plotted on a quantile plot. Patients were then categorisedcategorised into alta and baja using Insall-Salvati and Caton – Deschamps ratios^4,10^. Additionally, the distribution and values for the study population’s patella height less than the 5th and those at the 95th or 98th centiles were identified.

A comparison was made between the diagnosis of patella alta as determined by the Insall-Salvati ratios and the diagnosis as determined by the Caton-Deschamps ratios (patella alta, baja, or normal patella height). This aimed to confirm if these patients had retained the same diagnosis. This procedure was repeated for patella baja. The kappa statistic was calculated to check the inter-rater reliability of both ratios. Statistical analysis was conducted using STATA (version 18; StataCorp)^14^.

## Results

As seen in Table 1, there were 434 patients, and the mean age of the study population was 59. There were approximately equal numbers of patients in both genders, female (*n* = 207) and male (*n* = 227). Insall-Salvati and Caton-Deschamps ratios were calculated for the local population, as shown in Table 3. **The intra-class coefficient for the calculated ratios made by the two authors was 0.9.**

**Table 3:** Study population described using Insall-Salvati ratios and Caton-Deschamps ratios.

Ratio	Mean(SD)	Range
Insall-Salvati	0.85(0.16)	0.18–1.43
Caton - Deschamps	1.02(0.18)	0.16–1.54

**Fig. 2:**
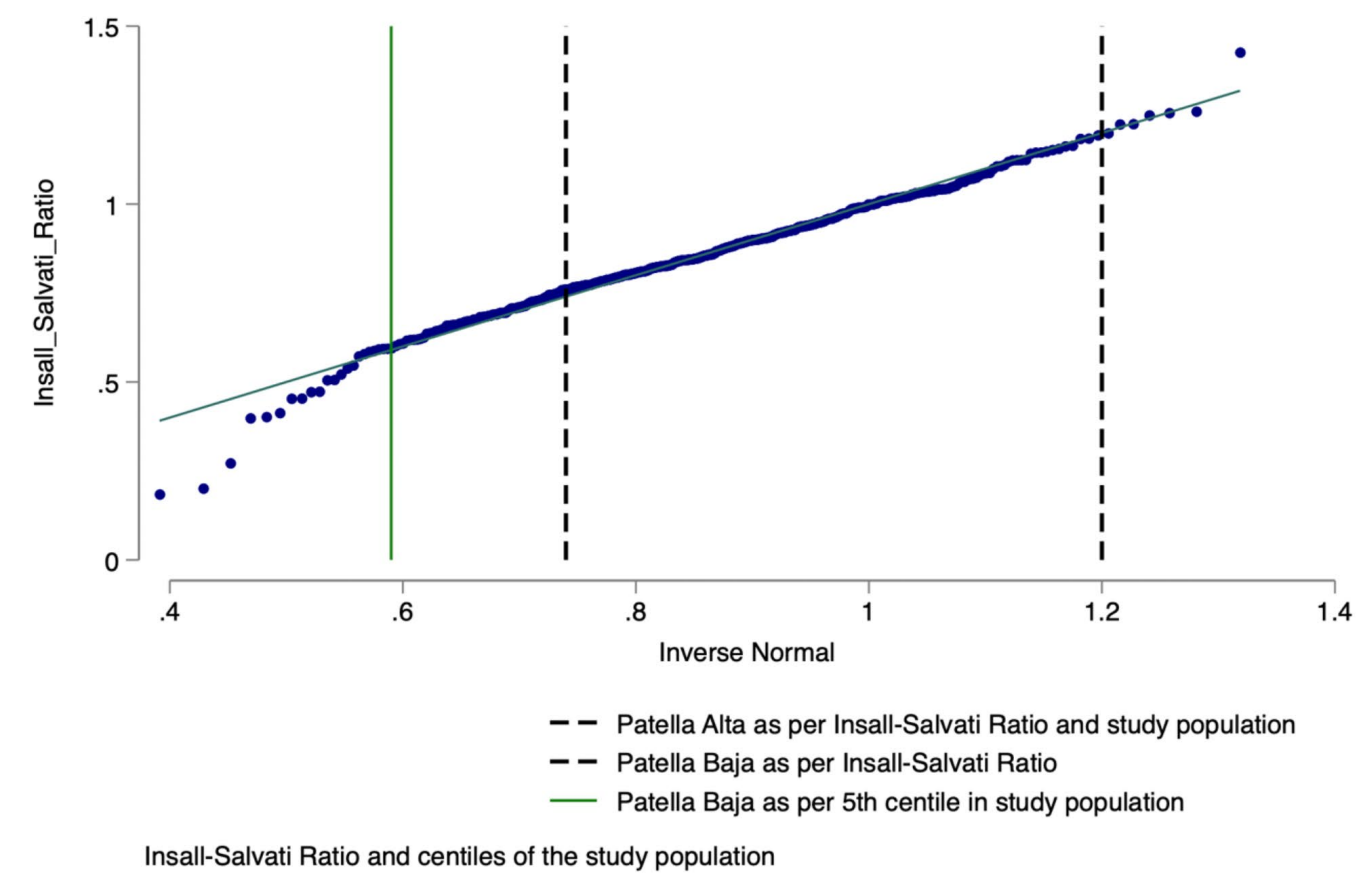
Local population distribution using Insall-Salvati ratio.

After calculating the Insall – Salvati ratio for each patient, the 98th centile in the study population was 1.2 (*n* = 10), which was equivalent to those classified as patella alta by Insall-Salvati, a ratio ≥ 1.2 (Fig. 2).

Meanwhile, the 5th centile in this study population was 0.59 (*n* = 19). Using the Chi-square test, in our study population, patients were being overdiagnosed with patella baja if dogmatically using the Insall-Salvati ratio, which classifies someone as patella baja with a ratio of ≤ 0.74 (*n* = 92) as compared to the local population’s 5th centile, 0.59 (*n* = 19), *p* = 0.01.

**Fig. 3:**
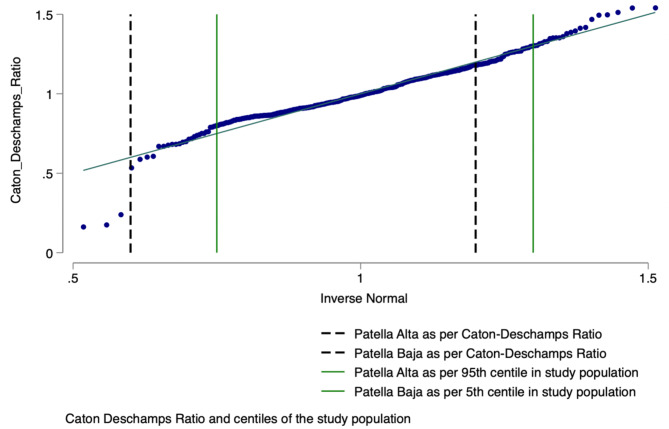
Local population distribution using Caton – Deschamps ratio.

As seen in Fig. 3, using the Caton – Deschamps ratio, the 95th centile in our population was 1.3 (*n* = 23), which was slightly higher than the patella alta ratio determined by Caton as a ratio ≥ 1.2 (*n* = 51). According to Caton – Deschamps ratios, patients were identified as baja if the ratio was ≤ 0.6 (*n* = 5). In our population, the 5th centile was 0.75 (*n* = 20). Using the Chi-square test, in our population, patients were being underdiagnosed if utilising dogmatically patella baja ratio as compared to the local population’s 5th centile *p* = 0.01.

The same can be said for patella alta; patients are being overdiagnosed if utilising dogmatically patella alta ratios are ≥ 1.2 as compared to the local population’s 95th centile, 1.3 (*p* = 0.01).

Only **three** patients were equally identified as patella alta in both Insall – Salvati and Caton – Deschamps ratios. For the patella baja category, the **five** patients classified as baja with the Caton-Deschamps ratio were equally identified as being baja using the Insall–Salvati ratio (same). The two ratios do not agree substantially more often than simply by chance, with a kappa coefficient of 0.01, indicating poor inter-rater reliability.

## Discussion

Insall- Salvati ratio was first described in 1971 using patients with radiological evidence of osteoarthritis and lateral radiographs with the knee in a semi-flexed position^4^. The total population in this case-series was 114 patients. Caton – Deschamps ratio was described in 1981 using 141 radiographs in flexed knee radiographs between 10’ and 80’^1,10^. The number of patients representing different ethnic groups is four times as high in our paper.

The Insall-Salvati ratio describes patella alta as greater than the 98% percentile. Using the same threshold as Insall et al., in our cohort, the population considered patella alta at the 98% centile had an equivalent ratio to what Insall-Salvati defines as alta. This means that in the case of alta, the central limit theorem is maintained whereby the sampling size in the original paper for alta was enough to represent the local population in this study as the limit of abnormality was equivalent to the alta in our 434 patient study^15^.

Insall and Salvati classify patients as patella baja if they have a ratio of less than 0.74, however, Insall does not specify if they are using the 5th centile as the lower limit of abnormality^4^. In our population, the 5th centile was 0.59, which means that patients with patella baja are being overdiagnosed in our cohort.

Regarding Caton-Deschamp’s original papers, the authors propose values based on the mean population of 141 subjects, extrapolating values for alta and baja from their sample’s mean population^1,10^. No details are given if the authors have used 98^th,^ 95th or 5th centiles as their limits of abnormality.

As of the 18th century, normal distribution curves have been used to understand population height, body mass index and even meteorological temperatures^16,17^. The median corresponds to the 50th percentile, the upper quartile to the 75th percentile and abnormalities lie in the tail end of the distribution^18^. For example, paediatric growth is based on growth centiles, the Centre for Disease Control and Prevention have released one growth chart reference population chart where the reference population was obtained from **five** national survey data sets between 1963 and 1994^19^.

Like growth, Patella height follows a normal distribution as shown in both Insall’s, Caton’s and our study population. However, both reference cohorts have utilised a small selected population not representative of the global population. This has been confirmed in several studies who have used patella alta indices published in 1971 and found that patients are being incorrectly classified^20–22^ Some of the reasons leading to this variability may be due to lifestyle factors in different countries such as India, Japan, China, South-East Asia and the Middle East^20^. Factors such as squatting and sitting cross-legged kneeling have been shown to stretch the patella tendon, affecting ratio calculation^20^.

All patients included in both Insall et al. and Caton et al., had a variety of knee pathology^1,4^. No details were given regarding the severity of osteoarthritis or previous history of patella pathology. These factors are essential, as osteoarthritis has been found to affect the length of the patella tendon^23,24^, therefore it is difficult to assume the indices will be reproducible in the healthy population.

A patient diagnosed as patella alta or baja with the Insall-Salvati ratio should be equally diagnosed as alta or baja using Caton – Deschamps ratio. From the 51 and **six** patients classified as alta using Caton and Insall’s ratios, only **three** patients were consistently labelled as alta. Five patients were classified as baja using Caton – Deschamps ratio and were equally labelled as baja using Insall-Salvati ratios, from an available 92 bajas.

Ultimately, one would expect a patient classified as alta with Insall-Salvati ratio would be the same patient classified as alta in Caton – Deschamps ratio. Otherwise, are we diagnosing or offering different treatments to patients depending on which ratio we use. This has been echoed in other papers, which show differing patella height classifications depending on the chosen index used^8^ .

With predictive algorithms generated by artificial intelligence we may be moving from the conventional approach of constructing and analysing large swaths of data to a future where machine learning methods are able to analyse, predict and accurately diagnose patients for all subpopulations^18,25,26^.

## Strengths & limitations

This is the first study to explore this concept, it is a healthy dataset representing different races and genders.

However, it is by no means a perfect dataset, where we lack equal representation from different ethnic groups. The mean age of this study population is 59.1 years, which is considerably older than the patient population typically affected by patellofemoral instability, limiting the generalisability of study results^27^. The use of CT to calculate tibial tuberosity torsion may have led to measurement error considering that the identification of the tibial tuberosity on CT may have been more challenging compared to MRI. Lastly, it is possible that some of the included patients had an unknown history of patellofemoral instability, leading to possible selection bias. However, the mean tibial tuberosity – trochlea groove (TT-TG) distance of all included patients (12.8 mm) is comparable to the mean CT measurement of TT-TG in groups without instability analysedanalysed in prior studies (12.6 mm – 12.7 mm) suggesting that the included patient population is representative of patients without patellar instability^18,20^. In this study patients were analysed with the knee in semi extended position whilst the original papers analysed knees in a 10’ – 80’ of flexion^1,4,10^. This study used CT images rather than radiographs, which has shown a significant correlation with previous studies^28^.

## Conclusion

Clinicians require upper and lower limits of normality in any type of measurement however caution needs to be exercised to understand the source of the reference population. Whilst we commend Insall-Salvati and Caton-Deschamps for conducting such high quality research in the early 1970–1980 s, we should strive to improve our measures by ensuring the indices are based on a large, more representative global population. Currently, Caton-Deschamps and Salvati’s ratios diagnose patients differently depending on the clinician’s ratio. The future seems likely to consist of a single set of charts; then we may begin to discuss the true definition of patella alta and baja with explicit reference values of what we identify is abnormal.

## Electronic supplementary material

Below is the link to the electronic supplementary material.

Supplementary Material 1

## Data Availability

The datasets used and/or analysed during the current study available from the corresponding author on reasonable request.
