# Supplementary material for: Patella height ratios diagnose the same healthy knees differently
Source: Sci Rep. 2025 Jan 2;15:89. doi: 10.1038/s41598-024-83663-2 (PMC11695827; doi:10.1038/s41598-024-83663-2)
Supplement: Supplementary file 1 — Supplementary Material 1 [file 41598_2024_83663_MOESM1_ESM.docx]

List of abbreviations:

Patellotrochlear index(PTI)

Stryker Orthopaedics Modeling and Analytics (SOMA)

Body mass index (BMI)

Tibial tuberosity –trochlea groove (TT-TG)
